# The Clinical Efficacy and Safety of Ertapenem for the Treatment of Complicated Urinary Tract Infections Caused by ESBL-Producing Bacteria in Children

**DOI:** 10.1155/2015/595840

**Published:** 2015-05-27

**Authors:** Ayse Karaaslan, Eda Kepenekli Kadayifci, Serkan Atici, Gulsen Akkoc, Nurhayat Yakut, Sevliya Öcal Demir, Ahmet Soysal, Mustafa Bakir

**Affiliations:** Department of Pediatric Infectious Diseases, Marmara University School of Medicine, 34890 Istanbul, Turkey

## Abstract

*Background*. Urinary tract infections (UTIs) are common and important clinical problem in childhood, and extended-spectrum-beta-lactamase- (ESBL-) producing organisms are the leading cause of healthcare-related UTIs. In this study, we aimed to evaluate the clinical efficacy and safety of ertapenem therapy in children with complicated UTIs caused by ESBL-producing organisms.* Methods*. Seventy-seven children with complicated UTIs caused by ESBL-producing organisms were included in this retrospective study, and all had been treated with ertapenem between January 2013 and June 2014.* Results*. Sixty-one (79%) females and sixteen (21%) males with a mean ± standard deviation (SD) age of 76.6 ± 52 months (range 3–204, median 72 months) were enrolled in this study.* Escherichia coli* (*E. coli*) (*n* = 67; 87%) was the most common bacterial cause of the UTIs followed by* Klebsiella pneumoniae* (*K. pneumoniae*) (*n* = 9; 11.7%) and* Enterobacter cloacae* (*E. cloacae*) (*n* = 1; 1.3%). The mean duration of the ertapenem therapy was 8.9 ± 1.6 days (range 4–11). No serious drug-related clinical or laboratory adverse effects were observed, and the ertapenem therapy was found to be safe and well tolerated in the children in our study.* Conclusion*. Ertapenem is a newer carbapenem with the advantage of once-daily dosing and is highly effective for treating UTIs caused by ESBL-producing microorganisms.

## 1. Introduction

Urinary tract infections (UTIs) are common bacterial infections in infants and children and have high prevalence and morbidity rates. Urinary tract infections are simply classified as acute pyelonephritis/upper urinary tract infection and cystitis/lower urinary tract infection. In a community with low antimicrobial resistance rates, UTIs in children older than 3 months can be treated with oral antibiotics for 7–10 days, for example, with cephalosporins or co-amoxiclav [1]. In conditions that oral antibiotics therapy is not possible (poor feeding, lethargy, toxic appearance, immunosuppression, etc.) intravenous (IV) antibiotics such as cefotaxime and ceftriaxone are appropriate therapeutic regimens for inpatient care. Parenteral antibiotic therapy may be stopped at 2nd–4th days and followed by oral antibiotics [1]. However, cephalosporins are mostly not effective for UTIs caused by ESBL-producing microorganisms, with the management of these infections being complicated by the increasing prevalence of these microorganisms in both healthcare-related and community-acquired UTIs. In our country, the data showed that prevalence of ESBL-producing bacteria in children is increasing, ranging from 20% to 54% [2, 3]. Kizilca et al. [4] reported that, in community-acquired UTIs caused by* E. coli* and* Klebsiella* species, ESBL productions were 41% and 53%, respectively.

Carbapenems, such as meropenem, imipenem, ertapenem, and doripenem, are one of the best antimicrobial therapy choices for infections caused by ESBL-producing microorganisms. Ertapenem is newer and has a narrower spectrum of activity than the others. It is effective against most Enterobacteriaceae and anaerobes, which are common causes of intra-abdominal infections, but it is less effective than the other carbapenems for* P. aeruginosa*,* Acinetobacter*, and Gram-positive bacteria [5]. The major benefit of ertapenem is that it has long half-life and it can be administered in a once-daily dose in contrast to three-four times daily for the other carbapenems.

Ertapenem is a beta-lactam antimicrobial agent that was licensed in the United States in November 2001 and in Europe in 2002. In addition, since 2005, it has been approved for use in children who are more than three months old with complicated skin and soft tissue infections, complex intra-abdominal infections, community-acquired pneumonia, UTIs, and acute pelvic infections [6].

In this study, we aimed to evaluate the clinical efficacy and safety of ertapenem in 77 children with complicated UTIs caused by ESBL-producing microorganisms.

## 2. Methods

Seventy-seven children aged three months to 18 years with UTIs caused by ESBL-producing organisms were included in this study. All had been treated with ertapenem between January 2013 and June 2014 in a tertiary care hospital. In this facility, the use of carbapenem for children is feasible after gaining the approval of the pediatric infectious diseases department.

In this retrospective study, the study participants were identified through the department's patient files archive, and their demographic information (age and gender), underlying diseases, clinical manifestations, and laboratory and radiological test results were evaluated.

Complicated UTI was identified by the presence all of the followings: (i) pyuria (a urinary white blood cell (WBC) count of >five bacteria per high-power field (HPF) for centrifuged urine); (ii) a positive dipstick for leukocyte esterase and/or nitrate; (iii) the presence of a recognized uropathogen at ≥10^5^ colony-forming units (CFU)/mL for midstream urine, ≥10^4^ CFU/mL for catheter urine, or >0 CFU/mL for suprapubic puncture urine; and (iv) the presence of two or more UTI symptoms such as fever, hypothermia, suprapubic tenderness, dysuria, urgency, or frequency [7]. The patients with UTIs who did not match these criteria were excluded from the study.

We used the Vitek 2 automated system (bioMèrieux) to identify the microorganisms and assess their susceptibility, and the antimicrobial susceptibility results and ESBL production were determined according to the guidelines of the Clinical and Laboratory Standards Institute (CLSI) [8]. In addition, all isolates were evaluated for ESBL production via the double-disk synergy test (DDST) using Mueller-Hinton agar.

When the patients were hospitalized, empirical antibiotic therapy was started depending on community or nosocomial urinary tract infection that was diagnosed. Cephalosporins were started for community-acquired complicated UTIs and meropenem was started for nosocomial UTIs, empirically. On the third day of empirical antibiotic therapy urine culture and antibiogram results were achieved and then changed to appropriate antibiotic. In this study, we focused on only patients treated with ertapenem. We administered ertapenem 30 mg/kg/day by dividing it into two intravenous doses.

## 3. Results

There were 61 (79%) females and 16 (21%) males with a mean age of 76.6 ± 52 months (range 3–204, median 72 months). We determined that 51 (66%) patients had an underlying predisposing factor for a UTI. Fourteen (18.2%) had a neurological (myelomeningocele with a neurogenic bladder) abnormality, 10 (13%) had vesicoureteral reflux (VUR), 10 (13%) had an anatomic anomaly (e.g., obstructive uropathy, bladder exstrophy, or a neurogenic bladder), six (7.8%) had bladder dysfunction representing with enuresis, four (5.2%) had undergone chronic hemodialysis, three (3.8%) had urolithiasis, and four (5.2%) had other anomalies, including two with cerebral palsy (CP), one with a malignancy, and one with congenital myasthenia gravis (Table 1). We accepted patients who had undergone chronic hemodialysis as secondary immunocompromised patients. The patients with underlying risk factors for UTIs were receiving prophylaxis including trimethoprim-sulfamethoxazole, nitrofurantoin, or amoxicillin. Ertapenem was initiated in all of the patients after the results of the microbiological cultures became available, and we determined that* Escherichia coli *(*E. coli*) (*n* = 67; 87%) was the most common bacterial cause of the UTIs.* Klebsiella pneumonia *(*K. pneumonia*) was identified as the source in nine patients (11.7%), whereas* Enterobacter cloacae* (*E. cloacae*) was the culprit in another patient (1.3%) (Table 2). All of the isolates were susceptible to the carbapenems including ertapenem, meropenem, and imipenem, but the ESBL-producing Enterobacteriaceae isolates were most frequently resistant to ampicillin (*n* = 75; 97.4%) and ceftriaxone (*n* = 75; 97.4%) followed by trimethoprim-sulfamethoxazole (*n* = 46; 59.7%), piperacillin-tazobactam (*n* = 42; 54.5%), gentamicin (*n* = 23; 29.9%), and nitrofurantoin (*n* = 16; 20.8%) (Table 3). The ESBL-producing* E. coli* was most often resistant to ampicillin (*n* = 65, 97%) and ceftriaxone (*n* = 65, 97%), but it was also resistant to trimethoprim-sulfamethoxazole (*n* = 39; 58.2%), piperacillin-tazobactam (*n* = 37; 55.2%), gentamicin (*n* = 22; 32.8%), and nitrofurantoin (*n* = 9; 13.4%). On the third day of ertapenem therapy, we obtained control urine cultures, and all resulted to be sterile. Clinical cure was accepted as the resolution of infection-related signs and symptoms after 48-hour onset of ertapenem therapy. And clinical cure was achieved in all patients. The blood culture results were available for all patients and all cultures resulted to be negative. None of the patients were bacteremic. The mean duration of ertapenem therapy was 8.9 ± 1.6 days (range 4–11), and we observed two drug-related adverse events (AEs), with one patient having a mildly elevated level of alanine aminotransferase and another patient developing a short-term maculopapular rash.

## 4. Discussion

Urinary tract infections caused by community-acquired and healthcare-related ESBL-producing* E. coli* and other Gram-negative bacilli have become widespread around the world since community-acquired ESBL-producing microorganisms were first discovered in 1998 in Ireland [9–12]. Carbapenems, such as meropenem, imipenem, ertapenem, and doripenem, are the most common antimicrobial agents used for treating several infections caused by ESBL-producing microorganisms because ESBLs are the enzymes which confer resistance to most beta-lactam antibiotics, including penicillins, cephalosporins, and aztreonam. Aminoglycosides can be used even after documentation of in vitro activity; however, the potential for emergence of resistance on treatment often limits their use. Furthermore, genes responsible for ESBLs are in close relation with resistance determinants to other antimicrobials (aminoglycosides, fluoroquinolones, and trimethoprim-sulfamethoxazole) [13, 14].

There are few reports regarding the clinical efficacy of the use of carbapenems for UTIs in children caused by ESBL-producing microorganisms. Ertapenem may be preferred over imipenem or meropenem because of its lower cost, feasibility for outpatient intramuscular therapy, and potential value for reducing carbapenem resistance in* Acinetobacter baumannii *(*A. baumannii)* and* Pseudomonas aeruginosa *(*P. aeruginosa*). In addition, there is also scarcity of reports concerning the clinical efficacy of ertapenem in the treatment of UTIs in children caused by ESBL-producing microorganisms. However, we believe that this particular carbapenem may be suitable for the first-line treatment of UTIs caused by these microorganisms in children.

The safety and efficacy of the use of ertapenem in children between the ages of three months and 17 years are based on evidence from well-controlled adult studies, pediatric pharmacokinetic data, and additional data from comparator-controlled studies that focused on pediatric patients [12, 15]. All of the patients in our study were at least three months old.

The duration of UTI therapy usually lasts from seven to 10 days; however, we suggest a longer course of therapy for children with an underlying predisposing factor for this type of infection. Unfortunately, the extended therapy necessitates longer hospital stays, exposure to more hospital-related infections, and higher treatment costs. Ertapenem can be given once a day intravenously in the hospital or intramuscularly as an outpatient, with the latter shortening the length of hospital stays. In turn, this decreases the costs and risks associated with the longer treatment.

Having an anomaly in the urinary tract increases the risk of UTIs [16], and predisposing obstructive abnormalities, for example, posterior urethral valves and ureteropelvic junction obstruction, as well as neurological abnormalities, such as myelomeningocele with a neurogenic bladder, and functional abnormalities, for instance, bladder dysfunction, may be anatomical in nature. In previous studies, VUR was the most common urological anomaly and the most common predisposing factor for UTIs in children [17, 18]. However, in our study, myelomeningocele with a neurogenic bladder was the most common underlying predisposing factor while VUR with the source was present in only 10 (13%) patients.

Ertapenem is primarily metabolized by the kidneys with minimal hepatic metabolism, which results in high antibiotic levels in the urine. The most common ertapenem-related AEs in previous studies were elevated hepatic transaminase levels (8.8%), diarrhea (5.0%), thrombophlebitis (4.5%), nausea (2.5%), and seizures (0.2%) [19], whereas the only two drug-related AEs that we observed were a mild elevation in alanine aminotransferase levels and a short-term maculopapular rash, both of which were reversible without having to discontinue the ertapenem therapy. Hence, ertapenem was well-tolerated in all our patients without any serious AEs.

Repeating the urine culture during the treatment of UTIs is no longer recommended [20], but, in our hospital, after the beginning of the therapy control urine cultures are obtained in clinical practice, especially in patients with underlying urinary anomalies. In this study, we obtained control urine cultures on the third day of the therapy, and all of the cultures resulted to be sterile with ertapenem therapy.

In addition, 66% of the patients in our study had underlying abnormalities that required at least ten days of treatment with hospitalization, but since the urine cultures were sterile on the third day of ertapenem treatment, the patients were then discharged and only needed single-dose intramuscular ertapenem maintenance therapy as outpatients for the next seven days.

Limitations of this study were as follows. (1) The data for episodes of UTIs was not given because of multicenter follow-up of patients. The patients' files in other hospitals were not available. For this reason, we did not give the episode data because of its unreliability. (2) We did not compare ertapenem with any other therapeutic agents in this study because of the retrospective study design. (3) We did not perform cost analysis of different antibiotic therapy regimens and their comparison with ertapenem therapy.

## 5. Conclusion

Ertapenem appears to be a good choice for first-line therapy for UTIs caused by ESBL-producing microorganisms in children. Not only did we find that it was an effective treatment option in this study, but also it was well tolerated by the patients. Furthermore, ertapenem has the advantage of shorter hospital stays and lower healthcare costs.

## Figures and Tables

**Table 1:** Predisposing factors for urinary tract infection, *n* (%).

Neurological (myelomeningocele with a neurogenic bladder) abnormality	14 (18.2)
Vesicoureteral reflux (VUR)	10 (13)
Anatomic anomaly (e.g., obstructive uropathy, bladder exstrophy, or a neurogenic bladder)	10 (13)
Bladder dysfunction representing with enuresis	6 (7.8)
Patients had undergone chronic hemodialysis	4 (5.2)
Urolithiasis	3 (3.8)
Others (malignancy, etc.)	4 (5.2)

**Table 2:** Distribution of the bacterial isolates, *n* (%).

*E. coli *	67 (87)
*K. pneumoniae *	9 (11.7)
*E. cloacae *	1 (1.3)

**Table 3:** Resistance rates of Enterobacteriaceae strains against antimicrobial agents, *n* (%).

Ampicillin	75 (97.4)
Ceftriaxone	75 (97.4)
Trimethoprim-sulfamethoxazole	46 (59.7)
Piperacillin-tazobactam	42 (54.5)
Gentamicin	23 (29.9)
Nitrofurantoin	16 (20.8)
Carbapenems (ertapenem, meropenem, and imipenem)	—

## References

[1] NICE (National Institute for Health and Care Excellence) (2007). Urinary tract infection in children: diagnosis, treatment and long-term management. *Clinical Guideline*.

[2] Çelebi S., Yüce N., Çakır D., Hacımustafaoğlu M., Öakaya G. (2009). Çocuklarda genişlemiş spektrumlu *β*-laktamaz üreten *E. coli* enfeksiyonlarında risk faktörleri ve klinik sonuçları, beş yıllık çalışma. *Çocuk Enfeksiyon Hastalıkları Derneği*.

[3] Demir N., Gençer S., Özer S., Doğan M. (2008). Genişlemiş spektrumlu *β*-laktamaz üreten gram negatif bakteri infeksiyonları için çeşitli risk fakörlerinin araştırılması. *Flora*.

[4] Kizilca O., Siraneci R., Yilmaz A. (2012). Risk factors for community-acquired urinary tract infection caused by ESBL-producing bacteria in children. *Pediatrics International*.

[5] UpToDate Database http://www.uptodate.com/contents/combination-beta-lactamase-inhibitors-carbapenems-andmonobactams?source=machineLearning&search=ertapenem&selectedTitle=5~36&sectionRank=1&anchor=H3#H3.

[6] Keating G. M., Perry C. M. (2005). Ertapenem: a review of its use in the treatment of bacterial infections. *Drugs*.

[7] Elder J. S., Kliegman R. M., Behrman R., Jenson H., Stanton B. (2007). Urinary tract infections. *Nelson Textbook of Pediatrics*.

[8] National Committee for Clinical Laboratory Standards (2000). *Methods for Dilution Antimicrobial Susceptibility Tests for Bacteria That Grow Aerobically*.

[9] Zilberberg M. D., Shorr A. F. (2013). Secular trends in gram-negative resistance among urinary tract infection hospitalizations in the United States, 2000–2009. *Infection Control and Hospital Epidemiology*.

[10] Paterson D. L., Bonomo R. A. (2005). Extended-spectrum *β*-lactamases: a clinical update. *Clinical Microbiology Reviews*.

[11] Ben-Ami R., Rodríguez-Baño J., Arslan H. (2009). A multinational survey of risk factors for infection with extended-spectrum beta-lactamase-producing enterobacteriaceae in nonhospitalizd patients. *Clinical Infectious Diseases*.

[12] Dalgic N., Sancar M., Bayraktar B., Dincer E., Pelit S. (2011). Ertapenem for the treatment of urinary tract infections caused by extended-spectrum *β*-lactamase-producing bacteria in children. *Scandinavian Journal of Infectious Diseases*.

[13] Fernández-Reyes M., Vicente D., Gomariz M. (2014). High rate of fecal carriage of extended-spectrum-*β*-lactamase-producing *Escherichia coli* in healthy children in Gipuzkoa, northern Spain. *Antimicrobial Agents and Chemotherapy*.

[14] Coque T. M., Baquero F., Canton R. (2008). Increasing prevalence of ESBL-producing Enterobacteriaceae in Europe. *Eurosurveillance*.

[15] Parakh A., Krishnamurthy S., Bhattacharya M. (2009). Ertapenem. *Kathmandu University Medical Journal*.

[16] Freedman A. L., Litwin M. S., Saigal C. S. (2007). Urinary tract infections in children. *Urologic Diseases in America. U.S. Department of Health and Human Services, Public Health Service, National Institutes of Health, National Institute of Diabetes and Digestive and Kidney Diseases*.

[17] Hansson S., Bollgren I., Esbjörner E., Jakobsson B., Mårild S. (1999). Urinary tract infections in children below two years of age: a quality assurance project in Sweden. *Acta Paediatrica*.

[18] Stokland E., Hellström M., Jacobsson B., Jodal U., Sixt R. (1998). Evaluation of DMSA scintigraphy and urography in assessing both acute and permanent renal damage in children. *Acta Radiologica*.

[19] Teppler H., Gesser R. M., Friedland I. R. (2004). Safety and tolerability of ertapenem. *Journal of Antimicrobial Chemotherapy*.

[20] Currie M. L., Mitz L., Raasch C. S., Greenbaum L. A. (2003). Follow-up urine cultures and fever in children with urinary tract infection. *Archives of Pediatrics and Adolescent Medicine*.

